# Grammatical Representations of the Verb Phrase in Mandarin Chinese: Evidence from Syntactic Priming in Five-Year-Olds

**DOI:** 10.3390/brainsci14111074

**Published:** 2024-10-28

**Authors:** Dong-Bo Hsu

**Affiliations:** Department of Chinese as a Second Language, National Taiwan Normal University, Taipei 10610, Taiwan; dhsu2@ntnu.edu.tw

**Keywords:** *aspectual -le*, five-year-olds, SVO-*ba* alternation, syntactic priming, verbal complement

## Abstract

**Background/Objectives:** Debates regarding how to represent verb phrases (VPs) consisting of the verb plus the complement and the aspectual marker -*le* in Mandarin Chinese remain an issue. **Methods:** Syntactic priming under a memory disguise paradigm was employed to investigate the issue using the SVO-*ba* alternation, where the SVO structure consists of a subject verb object, and the *ba* structure of a subject *ba* object verb, in five-year-olds (*n* = 216), an age with fully fledged grammatical knowledge but little interference from literacy. **Results:** The results indicate that both the complement and the marker -*le* should be represented in terms of phrasal rather than morphological structures. When -*le* is inflected to the verb alone, realization, which makes an event a fact, rather than completion, which makes an event finished, is accomplished. The event must be telicized to a state through a resultative complement to induce reliable production of the *ba* construction. The postverbal elements represent their own phrasal structure and challenge the verb-centered lexico-syntactic account because there are no additional representations left within a verb. **Conclusions:** More elicitations of the SVO than the *ba* invite future neurolinguistic explorations to disentangle the impacts of the frequency and thematic arrangement of agent and patient on grammatical representations cross-linguistically.

## 1. Introduction

The linguistic representations that underlie the ubiquitous use of language in daily life have long been an issue for researchers in language-related fields because knowledge of these representations is a way to understand the inner workings of the mind. Mandarin Chinese is no exception to this trend, as Chinese linguists have debates over how to represent the postverbal complement and inflected -*le* within a verb phrase (VP) [1,2,3,4,5,6,7,8,9,10,11] as to whether they are represented in terms of phrasal or morphological structures. The current study aims to employ the SVO-*ba* alternation in (1), where the SVO structure consists of the subject verb object in (1a), whereas the *ba* structure consists of the subject *ba* object verb in (1b), to investigate five-year-olds’ demonstrations of syntactic priming with this alternation to shed light on the debates of the inner representations of the VP. Traditionally, in the realm of linguistics, judgments of grammaticality play a significant role in addressing such issues. Grammaticality judgments typically occur when native speakers or language users are asked to judge prepared sets of sentences by rating them or providing judgments to determine whether the presented sentences are acceptable. Metalinguistic decision-making processes are inevitably involved, and the results tend to focus on knowledge that does not belong to the testees’ grammar. While the inability of the testees’ grammaticality judgment to determine the syntactic representations of the structures in question is often complemented by constituency tests, these tests have been found to be unreliable in determining linguistic structure, as they are likely to result in inconsistent constituent structures that the grammatical representations rely on. Branigan and Pickering [12] offered several examples to demonstrate how constituency tests may lead to contradictory and therefore unreliable results. For example, ellipsis and question–short answer tests often hint at the existence of a constituent in syntax; for example, in *I said he baked a cake and in fact he did so/What did he do?, Baked a cake* indicates a phrasal constituent. However, this is not the case when topicalization and it-cleft tests are applied, as in * I said he baked a cake and baked a cake he/*It is baked a cake that he, where * marks a lack of grammaticality [12] (p. 4–5). Following Bock’s pioneering works [13,14] using syntactic priming, which can target syntactic representations during language use, she and her colleagues reported that participants would reuse processed syntactic structures for subsequent target production without being explicitly aware of doing so. Such specific aspects of reuse of the processed structure without appealing to metalinguistic processes [12,15,16] can be preferred over grammaticality judgments and constituency tests, which may lead to inconsistent constituent structures for investigating grammatical representations. As the primary evidence employed to argue for grammatical representations within VPs relies on constituency tests and metalinguistic analysis, the advantage that syntactic priming can target potential syntactic representations during language production implicitly is believed to be promising in investigating the inner representations of VPs. The current study aimed to investigate the syntactic representations underlying the SVO and *ba* constructions illustrated below to shed light on issues and debates regarding linguistic representations of verb phrases in Chinese linguistics.
(1) a.Gongren gai-hao-le fanzi.

worker build-good-LE house
b. Gongren ba fanzi gai-hao-le.

worker BA house build-good-LE

‘Workers built (-complete) the house.’

The examples in pair (1) indicate that SVO and *ba* constructions are able to alternate [9,17] in the SVO-*ba* alternation, similar to the well-known dative and active–passive alternations in English. What concerns us is that these two types of constructions are actually able to be inflected alone, such as *gongren gai-le fanzi* vs. *gonren ba fanzi gai-le*, with the complement as a *VC*, such as *goren gai-hao fanzi* vs. *gongren ba fanzi gai-hao*, or the *VC-le* indicated in (1). Seemingly superficial and simple structures such as those represented above arouse debates on how grammatical elements are inflected after verbs are represented [1,2,3,4,5,6,7,8,9,10,11]. Since grammatical representations must be revealed and grammaticality judgments and constituency tests seem less likely to resolve issues involving these representations, the current study attempts to gain insight into how the grammatical elements inflected following the verb should be represented by testing Mandarin-speaking five-year-olds whose grammatical representations are considered fully grown [18] via syntactic priming of the SVO-*ba* alternation.

### 1.1. What (Morpho) Syntactic Representations Does Syntactic Priming Target?

Bock [13] demonstrated that when individual speakers heard and repeated dative alternations, which consisted of the prepositional dative *A rock climber sold some cocaine to an undercover agent* and the double-object dative *A rock climber sold an undercover agent some cocaine*, and active–passive alternation, which consisted of the active structure, *one of the fans punched the referee* and the passive structure, *the referee was punched by one of the fans*, they tended to reuse the repeated structure to describe the following target event, which was compatible with either structure under the guise of a memory task. For example, after they repeated a double-object sentence, they were more likely to use this structure to describe the event in which the man read a story to the boy, resulting in the use of *the man is reading the boy a story* to describe such an event. The sentence that individual speakers need to repeat is called a prime, and the subsequent event that they need to describe is called a target. This repetition of the structures is called syntactic priming. The example above indicates that syntactic priming occurs when there is no lexical overlap between the prime and the target. Bock [13] and Bock and Loebell [19] demonstrated that this syntactic priming operates on abstract and syntactically encoded phrasal levels that are not bound to specific lexical items. Bock [13] used both prepositional dative and double-object structures as primes. However, she particularly employed two types of prepositional dative structures as primes, namely, *to-* and *for-* prepositional dative structures, as *the secretary was taking a cake to her boss* and *the secretary was baking a cake for her boss*. On the other hand, the target pictures contained events that could be described only in terms of *to-* (but not *for-*) prepositional dative structures. She reported that both *to-* and *for-* prepositional dative structures induced relatively similar priming effects, suggesting that it is the prepositional phrase (PP), not particular function words such as *to* or *for*, that lead to syntactic priming. Bock and Loebell [19] provided more evidence to support the viability of this hypothesis via locative prepositional phrases (e.g., *the wealthy widow drove an old Mercedes to the church*), whose structure has been assumed to have a different nature in the noun phrase (NP) and PP, namely, the adjunct following the verb *drove* from the NP and PP and an argument following the verb, such as *give*, in the dative alternation. Despite these disparities in the natures of the NP and PP, locative prepositional structures are primed as much as their prepositional dative counterparts are.

The syntactic locus for the induction of syntactic priming was further strengthened in Hartsuiker and Westenberg [20] and Konopka and Bock [21], who reported that syntactic priming occurs when the pair of prime structures involves no discernable semantic difference. Hartsuiker and Westenberg [20] reported that primes of the auxiliary and main verbs, namely, *was stolen*, as *was gestolen* vs. *gestolen*, can alternate freely with each other in word order in Dutch and are assumed to be least involved in semantics-induced syntactic priming. Konopka and Bock [21] reported that word order variations within English phrasal verbs were sensitive to priming manipulations. Phrasal verbs (e.g., *pulled the sweater off* vs. *pulled off the sweater*) induced reliable syntactic priming. These studies showed that syntactic priming was sensitive to grammatical levels within phrases, but it seemed subject to one condition in which the elements within the verb phrase (VP) needed to be independent words but not morphological inflections. Pickering and Branigan [22] reported that regardless of what morphological forms, such as tense, number, and aspect features, were suffixed to the verbs (e.g., *the racing drivers were showing/showed/shows the torn overall to the mechanic*) in the prime sentences, all of the prime sentences induced syntactic priming of similar magnitude.

Pickering and Branigan [22] proposed a lexicalist approach that extended the model of lexical access developed by Levelt, Roelofs, and Meyer [23] to account for the occurrence of syntactic priming. They argued that syntactic priming targets phrase structure rules such as VP → V NP PP or VP → V NP NP for dative alternation in linguistics, which do not involve movement and syntactic representation of empty categories [12] and resemble non-transformational theories [24,25]. Therefore, each verb is represented in terms of a lemma, which is not a complete lexical entry because it does not encode semantic and phonological information but is associated with syntactic information such as number and gender. The lemmas of the verbs such as *give* or *show* are linked to the combinatorial nodes that resemble phrase structure rules such as NP-PP or NP-NP. Lemma and phrasal structures such as NP-PP or NP-PP are represented in terms of different types of nodes that are connected to each other by their grammatical properties. For example, *give* is a dative verb that can usually be used in the so-called dative alternation, where *give* can be alternated with the prepositional dative *the racing driver was giving the torn overall to the mechanic* or the double-object dative *the racing driver was giving the mechanic the torn overall.* Therefore, the verb *give* is represented as a lemma node that connects to both the prepositional dative phrasal structure and the double-object dative, i.e., two respective combinatorial nodes represented in terms of the NP-PP for the prepositional dative and the NP-NP for the double-object dative. When a prime sentence is processed, it activates the nodes of the verb lemma and the combinatorial nodes as well as the link between two nodes. Syntactic priming occurs when residual activation of these nodes is still operative for individual speakers’ subsequent target processing. However, it does not matter whether subsequent sentences contain identical or different forms, such as morphological suffixations, because the same lemma node is activated in both prime and target sentences. In other words, syntactic priming, as its name indicates, is sensitive to syntactic operations, namely, the phrasal structure, but inert to morphological structures within a word.

As appealing as this account may be, other factors may play a role in the induction of syntactic priming. Pickering, Branigan, and McLean [26] asked participants to complete four types of incomplete sentences in both written and spoken production: the prepositional dative (*the racing driver showed the torn overall*…), the double-object dative (*the racing driver showed the helpful mechanic*…), the baseline (*the racing driver sneezed very*…), and the shift-dative (*the racing driver showed to the helpful mechanic*…), which served as prime sentences. Once the participants completed these tasks, they were primed to that particular type of structure. Although the participants completed the primes of the prepositional dative and shift-dative, similar to how the order of the NP and PP reversed, their target completions in sentences such as *The patient showed*… were significantly different. While the prepositional dative induced syntactic priming, as many studies have reported, the shift-dative primes led to no priming, similar to the production following the baseline primes. On the basis of the lexicalist account of syntactic priming, there is supposedly a combinatorial node of PP-NP for shift-dative verbs similar to that of NP-PP, and their residual activation should induce syntactic priming. However, the results suggest that this is not the case for shift-datives. It is likely that construction frequency may play a role in syntactic priming [12,27] or that dative shift primes are represented at a level that is different from typical prepositional and double-object datives [28]. As a result, drawing upon syntactic priming’s characteristic sensitivity to phrasal structures but inertness to morphological structures in language production, syntactic priming is suitable to help shed light not only on grammatical representations of the postverbal elements inflected to verbs in the SVO-*ba* alternation but also on additional factors that may be involved in the production of these structures. On the other hand, the results obtained from investigations of the grammatical representations of the postverbal elements in the SVO-*ba* alternation may also test the claim that phrasal structures such as VPs can be represented only in terms of a single representation [26] or that there may be more than one representation within it [20,21]. Since the lexical-syntactic account is driven by a lemma of verb property, there are no dividable representations within a verb. If elements within a verb such as the postverbal complement and inflected -*le* can be represented in terms of more than one level with its own syntactic priming effect, then such a single-representation account is deemed untenable.

### 1.2. Syntactic Priming in Children’s Mandarin

Approximately two decades ago, Savage, Lieven, Theakston, and Tomasello’s [29] pioneering study employed syntactic priming as a window to explore the ontogeny of young children’s grammatical development and to examine whether their development of this syntactic representation reached the standards of the adult language. Most studies have reported findings that somewhat contradict Savage et al.’s [29] findings that children demonstrate lexically independent abstract knowledge no earlier than age 4. Researchers have reported that English-speaking children indicate the reliable emergence of abstract knowledge of active–passive alternation [30,31,32] and dative alternation [32,33,34]. Although Shimpi et al. [32] reported that abstract syntactic priming could occur only after three-year-olds heard and repeated the prime but would not occur if they only heard but did not repeat the prime, subsequent studies reported that children could demonstrate syntactic priming even when they did not repeat the prime sentences in dative alternation [33,34] or in active–passive alternation [31].

Studies of syntactic priming in children’s Mandarin have followed a similar trend as that in English-speaking preschoolers using the SVO-*ba* alternation illustrated above. Hsu [35] reported that syntactic priming occurred with this alternation after Mandarin-speaking three-year-olds comprehended and repeated primes. Three- to six-year-olds were able to demonstrate reliable syntactic priming after they comprehended the prime even without repeating it; the magnitude of the effects was similar across these ages [36,37], although the similar magnitude of syntactic priming effects throughout development differed somewhat from dative alternations [33,34] and active‒passive alternations [31] in English. The finding that Mandarin-speaking five-year-olds’ demonstrations of syntactic priming were similar in magnitude after they comprehended and produced the prime and after they comprehended the prime only suggested that they used a shared syntactic representation for language comprehension and production [36], similar to findings for adult English [22,27]. As a result, Mandarin-speaking five-year-olds serve as an ideal population to investigate how fully fledged grammatical representations with postverbal elements in the SVO-*ba* alternation in (1) are drawn upon during language processing with the least interference from literacy.

### 1.3. Issues Regarding Representations of the Postverbal Complement and -le Within a Verb

Syntactic priming with different syntactic representations permitted by the SVO-*ba* alternation in Mandarin Chinese not only allows us to evaluate the theoretical claim of accounts of lexico-syntactic representations but also serves as a window into what types of syntactic representations Mandarin speakers may encode during their language production. Because *ba* construction is often considered more constrained than the SVO structure is [4], issues regarding its compatibility with postverbal elements, whose capabilities make it possible to alternate them with SVO counterparts, initiate the following discussion.

The most apparent difference between an SVO and a *ba* construction in Mandarin Chinese is that the composition of the verb phrase in the *ba* construction must have some level of complexity, whereas the SVO does not necessarily require this level of complexity [4].
(2)Zhangsan chi(-le) yu.
Zhangsan eat-LE fish(3)Zhangsan ba yu chi*(-le).
Zhangsan BA fish eat*(LE)
‘Zhang ate fish’.

Grammatically, while both (2) and (3) have almost identical meanings, without the postverbal -*le*, the *ba* construction is not considered grammatical. However, it is less controversial whether the *ba* construction can be inflected by the postverbal -*le* and by an additional insertion of a complement. This is often another verb but is grammaticalized, denoting resultative meaning and forming the *VC-le* structure illustrated in (4). However, the issue of whether the postverbal element can be inflected by the complement alone without the -*le* marker leads to controversy. Huang and Yang [38] claimed that the *ba* construction can be considered grammatical only when the postverbal C(omplement) elements are inflected further by the -*le* marker.
(4)Zhangsan ba yu chi-wan*(-le). by Huang and Yang [38]
Zhangsan BA fish eat-finish-*(LE)
‘Zhangsan finished eating fish’.

Example (4) indicates that the inflection of -*le* must be required and must follow the (C)omplement; otherwise, the *ba* construction would be deemed unacceptable. Nevertheless, most verbs are compatible with the *ba* construction even when they are only inflected by the complement without the -*le* marker. You [39] paired the *ba* construction with -*le*, a complement, and a complement plus *-le,* and asked native Mandarin speakers to judge the grammaticality of these pairings. That study reported that many verbs with these combinations were scored greater than 4 on a 5-point scale. In other words, they were acceptable to native speakers, including the pairings of the inflections of complement alone to the verbs in the *ba* construction. The unacceptable sentence in (4) marked with an asterisk without the -*le* is supposed to be acceptable. Acceptable sentences with three different combinations were selected to constitute the primary materials in the study.

#### 1.3.1. The Postverbal -le

Since Chao [1], the postverbal -*le* has generally been considered a morpheme that expresses completed action [11]. The following pair is compared:
(5)a.Zhangsan mai-le wu-ben shu.

Zhangsan sell-LE five-CL book

‘Zhangsan sold five books.’
b.Zhangsan mai wu-ben shu.

Zhangsan sell five-CL book

‘Zhangsan sells/sold five books.’

Contrasting the presence and absence of -*le* indicates that when postverbal -*le* is present, the action of selling is completed, whereas when -*le* is absent, it may be only a description of a selling act, which may mean that *Zhangsan* may sell five books but may not necessarily have completed the action of selling. However, this semantic analysis has been challenged and rejected by Liu [5]. Interpretations of the postverbal -*le* are ambiguous and depend on the properties of the verbs with which -*le* may co-occur [7]. Liu [5] showed that sentences with adjectives were also compatible with -*le* and that verbs inflected with -*le* did not necessarily denote a complete state to argue that the semantic interpretation of postverbal -*le* should not be analyzed as a marker for completion.
(6)a.Ta gao-le wu gongfeng.

he tall-LE five centimeter

‘He has grown five more centimeters in height.’
b.Wo kan-le yi-ben shu.

I look-LE one-CL book

‘I read a book but I may not finish reading it.’

From (6a), we know that the mentioned person could or could not continue to grow in the future, and the information obtained from the sentence did not signal completion. It has a similar interpretation to (6b) on the basis of the translated gloss. As a result, the function of the postverbal -*le* should indicate that the action ‘expressed by the verb’ or the state ‘expressed by the adjective’ has become a fact. To strengthen her argument, she contrasted the verb to be inflected with *-wan* ‘finish/complete’ and -*le*.
(7)a.chi-wan cai juede you dian xiengwei.

eat-finished only feel have a little salty

‘Only after having finished the meal did I feel somewhat salty.’
b.chi-le cai juede you dian xiengwei.

eat-LE only feel have a little salty

‘Only when I ate, I felt somewhat salty.’

The contrast indicated that in (7a), only after the subject finished eating the meal did he realize that it was salty. In contrast, the subject did not need to finish the meal and perhaps ate only one or two bites before realizing that the meal was somewhat salty. Liu used the sentences to corroborate his claim that -*le* is only used to signal realization of the verb or the adjective that it co-occurs with becoming a fact and that postverbal -*le* is unrelated to completion.

Shi [7] argues that -*le* signals completion with bounded predicates, whereas with unbounded predicates, it signals realization that is seen as started and ongoing. The following pair of sentences is compared:
(8)a.chi-le yi-zhi ji cai juede you dian xiengwei

eat-LE one-CL chicken only feel have a little salty

‘Only after I had eaten a chicken did I feel it was salty.’ *Completion -le*
b.chi-le ji cai juede you dian xienwei

eat-LE chicken only feel have a little salty

‘Only when I ate (some) chicken did I feel it was salty.’ *Realization -le*

‘Only after I had eaten the chicken(s), I felt it was salty.’ *Completion -le*

When the formation of the predicate depicts a discrete and/or specific entity, it becomes bounded. Therefore, -*le* is interpreted as a completion marker, as shown in (8a). On the other hand, when the formation of the predicate depicts a nonspecific entity, as (8b) shows, it becomes unbounded; therefore, -*le* is interpreted ambiguously as either a realized state or a completed marker.

Sybesma [9] argues that all postverbal -*le* is ultimately interpreted as a certain state of realization, which he termed realization *le.* However, whether it is interpreted as a completion marker, which he called *the end point*, depends on the predicates themselves and the composition of the predicate and its following entities [7]. He specifically noted that predicates that signal realization are forced to further signal a completion interpretation, a view held by researchers of the *ba* construction [4,40,41,42]. However, a somewhat different description is presented in Huang, Li, and Li, [17] with respect to the *ba* construction, as follows:
(9)a.Ta ba shu kan-le. [9] (p. 146)

he BA book read-LE.
b.Ta kan-le shu.

he read-LE book

‘He read the book.’

Although he acknowledged that the interpretation of (9b), which is also (6b), would assert the existence of the subject reading a book or the realization of the subject reading a book, the sentence goes one step further to force the complete reading. This reading is forced in the *ba* construction, as indicated in (9a).

His claim of -*le* as a predicate that is represented in terms of small clause analyses, which consist of a subject and predicate, differs from the general understanding that the postverbal -*le* is a morphological marker that may occupy the Asp(ect)P [2,43]. Since every -*le* can always be a realization *le* but not necessarily an end point *le*, the two layers of interpretations are represented in terms of two different types of small clauses. These are termed XP, which is headed by the realization *le* meaning ‘realized’, and YP, which is headed by the end point *le*, meaning resultative, as illustrated below.
(10)
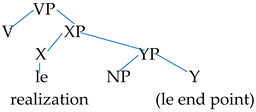


As the former discussion argues, realization *le* is always present and is predicated by another small clause, namely, YP. YP is responsible for results denoting interpretation, which may sometimes be realized as a phonologically empty element because the resultative interpretations are not always manifested in the SVO construction but are necessarily manifested in the *ba* construction. The end point *le* in the YP heads the YP and predicates the NP. The postverbal-*le* constitutes its own syntactic representations with different semantic interpretations.

#### 1.3.2. The Complement and -le Following the Complement

With respect to the inner structure of the verb + complement that allows the SVO-*ba* alternation in the current study, unlike the postverbal -*le*, there is consensus that the interpretation of the VC is primarily a resultative predicate where the first element indicates causal activity and the second element indicates the result. What stimulates the debate is the internal morphosyntactic representation.

Chao [1] analyzed these compositions as resultative compounds; that is, their composition is derived from a morphological process that combines two seeming words into a larger word (i.e., compound words); this analysis has been endorsed by other Chinese linguists [3,4,6]. In contrast to this morphological point of view, Teng [10] argues that this composition is, in fact, a phrasal structure that mirrors English verb‒particle phrases such as ‘look up’ and ‘take away’ and allows certain degrees of freedom within the two words. These compositions can include other elements, such as *de* ‘be able to’ and *bu* ‘unable to’, resulting in *V + de + C* or *V + bu + C*, although the permitted insertions are not fully productive. Sybesma [9] employed the small clause analysis developed by Hoekstra [44] to articulate that the composition of VC is a small clause, which is understood as the degree to which the matrix verb, namely, V, extends.
(11)a.Gongren gai-hao fanzi.

worker build-good house(s)

‘Workers built a house/houses (well).’
b.Gongren [_VP_ gai [_ExtP_ Ext^0^ [_SC_ fanzi hao]]]

To avoid excessively technical details and to focus on the inner structure of the VC, (11b) indicates that the postverbal noun phrase (NP) fanzi ‘house’ is the subject of the small clause and that the resultative complement hao ‘good/well’ serves as the predicate of this NP in the small clause. Through several movements, such as head movement, the resulting surface structure is what we obtain from (11a).

In Mandarin, (11a) can be further inflected with -*le*, resulting in (12).
(12)Gongren gai-hao-le fanzi.
worker build-good-LE house(s)
‘Workers built a house/houses (well).’

Sybesma [8] suggested that the postverbal -*le* may be the only predicate in Chinese that is inherently telic, which denotes a clear end point. In contrast to the resultative, such as hao ‘good/well’, which depicts the content of the result, -*le* not only telicizes the matrix event as the complement but also makes the event a state, which is lacking among other complements. In other words, while all of the sentences are grammatically formed, -*le* has an additional function that makes the matrix event a state.

### 1.4. The Current Study

In light of the literature on syntactic priming [14,22], different grammatical representations can lead to different levels of syntactic priming [20,21]. One of the most salient findings on the basis of Pickering and Branigan’s [22] work suggested that morphological representations such as aspect and tense are less likely to lead to syntactic priming. If the postverbal -*le* is presented as a suffix that occupies the ASP(P) [2,43], the SVO-*ba* alternation, where both of the structures are inflected with -*le* alone, should exhibit a priming level similar to its counterpart of the structures that are inflected with the VC-*le*, which has been demonstrated to have reliable syntactic priming in Mandarin-speaking preschoolers [35,36,37]. On the other hand, if the postverbal -*le* projects its own syntactic representations, each of which represents the realization, namely, realization *le*, and completion, namely, end point *le* [9], this SVO-*ba* alternation may lead to different magnitudes of syntactic priming from its VC-*le* counterparts, as -*le* serves a predicate that projects its own phrasal representation. Furthermore, since the postverbal -*le* is ambiguous when it is inflected with the SVO construction while this ambiguity disappears when it is inflected with the *ba* construction, it is likely that levels of ambiguity may exert an effect on syntactic priming [45].

Similarly, debates on the inner structures of the VC composition allow us to speculate that if VCs are, in fact, resultative compounds, which are morphological structures, and if all of these morphological structures eventually link to the syntactic structure and become a verb, it is likely to exhibit a similar syntactic priming effect to its VC-*le* counterpart. In contrast, if VC is itself a syntactically represented small clause, as treated by Sybesma [9], this additional layer of grammatical representation of small clause analysis may alter the magnitude of syntactic priming effects, which is expected to be different from either the -*le* or *VC-le* primes.

## 2. Materials and Methods

### 2.1. Participants

The experiments initially recruited 278 five-year-olds between the ages of 54 months and 66 months on the basis of the following criteria: (i) a predominantly monolingual Chinese language environment with less than 30% exposure to a language other than Mandarin Chinese (plus Taiwanese) and (ii) no history of medical conditions that would affect typical language development. While the children provided oral agreement to participate in the experiments after their parent’s consent, 5 children from the first experiment (*VC-le* experiment), 6 children from the second experiment (*le* experiment), and 6 children from the third experiment (VC experiment) refused to participate in the second stage of the experiments (see Section 2.3. Procedure below). These patients were excluded from further data analysis. Furthermore, to obtain sufficient trials for subsequent data analysis, children who produced > 50% of other utterances were excluded from further data analyses. This cutoff criterion was adopted because it would provide comparable ‘other’ rates to those [35,36,37] that have reported reliable syntactic priming in child Mandarin. This decision resulted in the exclusion of 5 children in the first experiment, 6 in the second experiment, and 34 in the third experiment. Therefore, 72 children were included in each experiment in an attempt to have a significance level of 0.05, with an effect size of 0.15, and a power of 0.8. The first experiment included 38 girls, the second experiment included 37 girls, and the third experiment included 38 girls for a total of 216 participants in the final data pool for the analysis of the syntactic priming of the SVO-*ba* alternation. Although all the participants spoke Mandarin as their dominant language, 31 participants in the first experiment, 29 in the second experiment, and 31 in the third experiment were bilingual in Mandarin and Taiwanese, but they interacted with their teachers primarily in Mandarin both in kindergarten and at home. The parents of the bilingual children reported in a background questionnaire that their children were dominant in Mandarin but that they could interact with their grandparents in simple Taiwanese and had little difficulty understanding Taiwanese. All the children’s parents were considered to have comparable socioeconomic statuses in that they were middle class or above.

### 2.2. Design and Materials

The entire experiment consisted of three subexperiments that primarily differed in their morphological forms, namely, *-Cle*, -*le*, and *C*, accompanied by the main structures of the SVO and *ba* constructions. From this perspective, the experiment employed a mixed design. The inflectional form was a between-participant variable with three levels: *VC-le*, *V-le*, and *VC*. The prime structure was manipulated as a within-participant variable. Each child heard both the SVO construction and the *ba* construction with fillers interspersed between the SVO and the *ba* constructions, i.e., a nonblocked design with fillers. As a result, for each subexperiment, the prime structure was treated as a within-participant variable. The experiment adopted a memory disguise paradigm that was initially employed in Bock’s series of studies in adults [13,46], where the participants reviewed prepared items by either watching pictures or listening to sentences and were asked to try their best to memorize these reviewed items because their recognition memory was tested in the second stage of the study. The reason it was called a memory disguised syntactic priming task was that in the second stage, while the participants were required to provide recognition memory responses to each item they encountered, they needed to repeat the sentence they heard, which was actually the prime sentence. After they provided an answer, they also needed to provide a recognition memory response to the paired picture and then use one sentence to describe the event in the picture, which was actually the target. On the basis of this design, the participants were led to believe that they were undergoing a memory experiment but not a priming experiment. The reason for employing this paradigm was that most syntactic priming in child language uses a dialog-like paradigm to investigate syntactic priming. Although it may be more suitable for preschoolers because it can be implemented more directly and in less time, it may have specific effects on syntactic priming [47]. Since five-year-olds were tested, who had conditions more comparable to those of adults and for whom a more syntactic focus was examined in the current study, this memory disguise paradigm was adopted.

Two experimental lists were prepared to counterbalance the study for each subexperiment. List 1 began with an SVO construction (SVO-first list), and List 2 began with a *ba* construction (*ba*-first list). The children were evenly distributed across the two lists. In addition to the experimental lists, a practice list was prepared. This practice list was administered prior to the experimental list so that the experimenter and the children could ‘warm up’ for the later study.

Each experimental list contained 24 experimental animations and 24 filler animations. The animations were created via Adobe Flash Player 9 (Taiwan). All experimental animations denoted transitive events that indicated a clear completion state and could be described via an SVO structure or an SbaOV structure. For example, an animation that showed a tiger pushing the rock away could be described via an SVO structure, as in Example (1a), or a *ba* construction, as in Example (1b). These verbs were employed as target animations. The verbs used in the event were compatible with each of the types of inflectional forms tested in this study, as indicated by a grammatical norming study with an acceptability score greater than 4 out of 5 evaluating scales for each verb [39]. The 24 verbs that were used to construct the events were *tu* ‘spit’, *wen* ‘ask’, *gai* ‘cover’, *jien* ‘build’, *tou* ‘steal’, *zhua* ‘scratch’, *jia* ‘add’, *jie* ‘catch’, *jie* ‘lend’, *jiao* ‘teach’, *xuan* ‘choose’, *shou* ‘collect’, *ti* ‘kick’, *na* ‘take’, *tuei* ‘push’, *jien* ‘cut’, *diou* ‘throw’, *bao* ‘hug’, *shao* ‘burn’, *si* ‘tear’, *chuan* ‘wear’, *kao* ‘bake’, *he* ‘drink’, and *zhai* ‘prick’. A full list of the 24 target events used to construct the animations is shown in Appendix A. Twelve target animations were paired with the SVO primes, and twelve target animations were paired with the *ba* construction primes. The verbs used to construct the event embedded in the prime sentences were *chi* ‘eat’, *huan* ‘exchange’, *mai* ‘buy’, *kan* ‘see’, mai ‘sell’, *chai* ‘disintegrate’, *zha* ‘explode’, *du* ‘read’, *zhuan* ‘hit’, *guan* ‘close’, *chuei* ‘blow’, *qian* ‘rob’, *dai* ‘wear (watch’), *la* ‘pull’, *tou* ‘tow’, *bang* ‘tie’, *ren* ‘throw’, *chong* ‘flush away’, *ba* ‘pull out’, *xi* ‘wash’, *ban* ‘move’, *tsai* ‘step on’, and *yao* ‘bite’. Similarly, 12 prime sentences were paired with the SVO primes plus the morphological forms required in the subexperiment, and 12 prime sentences were paired with the *ba* construction primes plus the required inflectional forms. The sentences used to make the target animations are listed in the Appendix A. The twenty-four filler animations allowed intransitive descriptions. Each animation involved an agent who performed a self-initiated action or an event that could be described via the intransitive structure of Mandarin. For example, one intransitive animation showed a cat standing on a chair. The filler sentence was also made using verbs that involved only 1 participant. All of the prime and filler sentences were recorded on an iPhone 13 and saved as audio files to be played for the experiments. These fillers, including sentences and animations, were interspersed with SVO-*ba* sentences in a transitive–intransitive order. The arrangement of fillers was fixed in Lists 1 and 2. The prime sentences were randomized and then paired with the target animations.

All of the prime sentences and the filler animations were placed in the first stage for the child participants to watch and listen to. They were told that their memory would be tested later regardless of whether they had watched the animation or listened to the sentences. During an experimental trial, the experimenter played a prime sentence, and the children answered whether they had heard the sentence before. The experimenter then presented the target animation, and the child uttered a one-sentence description. After the description, the children provided their response to whether they had previously watched the animation. The target events were events that five-year-olds were familiar with on the basis of some of their caregivers’ and kindergarten teachers’ reports. The lengths of the prime sentences were kept constant by using disyllabic noun phrases as agents and patients. The difference in the length of the sentences was the addition of the *ba* word in the *ba* construction and different types of morphological forms, which had systematic lengths for each form in the prime sentences.

The practice list consisted of 2 sets of practice trials, each including four dative prime sentences and four animations that described a transferal event. Both involved an agent/doer, a theme/object, and a recipient/receiver performing a transferal event. One set functioned as the trial in the learning stage, and the other set served as the test trial. Within the 2 sets of trials, 2 prime sentences and 2 animations overlapped, whereas the remaining 4 were different.

### 2.3. Procedure

Because the experimental materials were expected to be somewhat longer for preschoolers and were disguised in a memory task, two experimenters conducted the experiment. Each experimenter was paired with a child during the experiment. The experimenters were allocated two spaces: one dedicated to the experiment and another depending on the arrangement of the kindergarten or the lab. The first experimenter initiated the practice trials and stage I (the memory stage) and then proceeded to the first 10 trials in the experimental list with a paired child. The other experimenter watched Japanese cartoons with another child in another room/space (after the first experimenter finished the first part of the experiment) so that the two experimenters did not interfere with each other. The children were tested individually. The procedures were as follows: the first pair of the experimenter and child who conducted the first part of the experiment → they went to another room to see a Japanese cartoon → the second pair of the experimenter and child entered the experimental room → the second pair finished the first part of the experiment → the second pair came to the room for the cartoon → the first pair returned to the experimental room and finished the experiment → the first experimenter returned to the cartoon room to watch the cartoon with the third child → the second pair returned to the experimental room and finished the experiment → the second experimenter returned to the cartoon room to watch the cartoon with the fourth child → the first experimenter and the third child came to the experimental room, and then the entire procedure was repeated.

When they entered the room, the first experimenter asked the child if s/he would like to play a ‘how much you can remember’ game with her. The experimenter familiarized the child with the practice trials using the prepared dative events and asked him or her to listen to and watch the sentences and animations that the experimenter played. The experimenter subsequently played another set of materials to practice the memory test task. After the experimenter played the sentence, she asked the child whether s/he had previously heard the sentence. After the child provided a response, the experimenter played the animation and asked the child to use one sentence to tell her what happened in the animation. After the child provided a description, the experimenter asked the child whether she or he had seen this animation before. After the response, another trial continued until the end of the preparation process. Regardless of whether the child provided the correct memory response, the experimenter nodded and said ‘very good’. If the child forgot to provide a response, the experimenter reminded him or her to do so. After the practice trials, the experimenter told the child that the upcoming game would be more challenging because it would include more sentences and more animations. The experimenter asked the child to listen to the sentences and watch animations more carefully. The experimenter told the child that s/he had to pay attention to the materials and did not need to provide a response until the child was asked to do so. After the experimenter finished playing the set of prime sentences and filler animations, she told the child that it was time to see how much he or she remembered. When the experimenter played the sentence, the child needed to tell her if s/he had heard the sentence earlier. The child used one sentence to describe what happened in the following animation and then told the experimenter if she or he had seen the animation before. Around the 3rd trial, the child no longer needed prompts and automatically provided memory responses. However, if the child forgot, the experimenter reminded him or her to provide a response. The procedure was repeated until the 10th trial in the corresponding list. The experimenter then told the child that they would take a break to watch a Japanese cartoon and would continue the game after they watched the cartoon. The second pair then entered the room, the procedure was repeated for the first half of the experiment, and the participants were sent to the room for the cartoon. The first pair returned to the room to finish the experiment, and then the second pair entered the experimental room and finished the experiment. After the child finished the experiment, s/he received a packet of stickers.

### 2.4. Coding and Scoring

The five-year-olds’ animated descriptions were coded according to their syntactic structures. Since this study specifically investigated the influences of inflectional forms embedded within prime sentences on syntactic priming, two coding schemes, i.e., lax and strict coding schemes, were adopted. The lax coding scheme coded a description as a prime response as long as it was a grammatical structure that conformed to the general structure of the prime sentence regardless of its specific inflectional forms, with the constraint that for the SVO structure, at least the perfective particle must be present. To have a comparable coding scheme for the two structures, since the *ba* construction must have at least a level of complexity such that the verbal structure must be inflected with the perfective particle—while the SVO structure is more liberal in this respect—the SVO structure must be at least inflected with the -*le* marker to be included as a structure and considered primed or not primed. For an SVO structure that was not at least inflected with the marker -*le*, the response would be considered ‘other’. Therefore, if the prime sentence had a structure of Subject Verb-C(omplement)-*le* Object and the structure of the response of the animation description was Subject Verb-*le* Object in that order, then this trial was laxly coded as a primed trial and was recorded as 1. However, if the response was subject verb object in that order without a postverbal -*le*, then this trial was coded as an ‘other’ trial. To be coded as a primed trial in the strict scheme, the response of the target animation must have a structure with the same inflectional form depending on the conditions of the inflectional forms in question that match the prime structure. Following the example above, the structure of the target description should be the structure of Subject Verb-C(omplement)-*le* Object to be coded as a primed trial, namely, 1. If they shared the same syntactic structure but differed in the inflectional forms, that trial was coded as a nonprimed trial, namely, 0, which was coded as a prime trial as 1 in the lax coding schema. Sentences that did not conform to these syntactic descriptions, such as descriptions that had the addition of *zai* or *zhe* ‘-ing’ or sentences with no subjects or no objects, were coded as ‘other’. Trials were excluded and treated as ‘other’ trials if the children repeated the experimenter’s verb to describe the target animation that immediately followed. The data were coded by a trained coder. Data from twenty-four randomly selected children (eight for each subexperiment) were given to another trained coder for independent coding on the basis of the previously described coding schema. Disagreements were resolved by the author. The same coding procedures were applied to all the experiments in this study. The intercoder reliability rate for the three subexperiments was 96% (Cohen’s *k* = 0.95, *p* < 0.001).

## 3. Results

The number of responses and percentages of the SVO, *ba* constructions, and ‘other’ utterances in each priming condition across the three subexperiment groups that used the inflectional forms *-VC-le*, -*le*, and *-VC*, respectively, in both the SVO and *ba* constructions for the two coding methods are displayed in Table 1.

Table 1 indicates that when the methods of lax and strict coding were contrasted, different patterns for syntactic priming were revealed on the basis of the morphological forms embedded in the prime structures. Syntactic priming was generally displayed in both prime structures but not in the *ba* construction when it was inflected with VC forms when lax coding was applied to the data. In contrast, the number (and percentage) of priming trials generally decreased, and syntactic priming showed a three-way contrast for each of the three inflectional forms when strict coding was applied. When both prime structures were inflected with the *VC-le*, syntactic priming still reliably occurred. Nevertheless, when the prime structures were inflected with -*le*, only the SVO structure, not the *ba* constructions, showed syntactic priming. The sharply decreased number of responses indicated that a large proportion of the production of the *ba* construction was not in the -*le* form but rather in the other two forms. When the two structures were inflected with VC, neither of them showed syntactic priming effects, and more other types of utterances were obtained.

**Figure 1:**
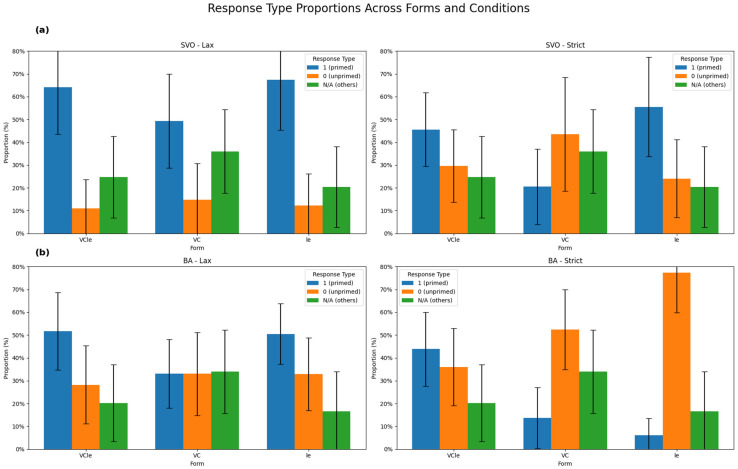
(**a**) The upper panels show the proportions of the SVO, *ba*, and other utterances produced when three types of inflectional forms in the SVO construction were used as primes in two coding methods; (**b**) the lower panels show the proportions of the SVO, *ba*, and other utterances produced when three types of inflectional forms in the *ba* construction were used as primes in two coding methods.

Figure 1 visualizes the priming effects when the SVO and *ba* constructions were inflected by the three different inflectional forms when they were coded strictly and loosely, respectively.

SVO primes demonstrated syntactic priming when lax coding was applied. However, when strict coding was applied, the participants seemed reluctant to produce SVO structures in terms of the VC form as shown in Figure 1a. Consequently, the SVO structure was primed in lax coding but not in strict coding when the prime was inflected with VC. On the other hand, this pattern of production showed that to reliably exhibit syntactic priming in production, the inflectional element in the SVO prime had to include a postverbal -*le*.

The results with respect to the *ba* construction in the figure indicate that when lax coding was applied and when the *ba* construction was inflected with the complement only, syntactic priming did not occur. The pattern shown in the strict coding indicated that although the *ba* construction could be generally applied after the *ba* prime was inflected with the *postverbal* -*le* in the lax coding, it primed the participants to produce forms other than *ba* + V-*le* as shown in Figure 1b. Therefore, when strict coding was applied, the V-*le* form did not actually induce reliable syntactic priming. The pattern suggested that although the *ba* construction can be primed when it is only inflected with -*le* in lax coding, a large proportion of the participants’ actual production of the *ba* constructions after the -*le* prime involved a complement, not just the pure form of -*le*.

The five-year-olds’ demonstration of syntactic priming with respect to these two coding schemes was subsequently investigated. The results of each subexperiment, where the prime structures inflected with *VC-le*, -*le*, and *VC* were reported via various logit mixed effect models, fitted to the data to account for the analysis of binary response variables while simultaneously considering both the random effects of the subject and the items [48,49,50]. All of these models were calculated via the glmer function of the lm4 package in R (2024.09.0 + 375). The factor labels of the structure and order were treated as a factor variable, and the factor of structure was centered prior to analysis to result in a mean of 0 and a range of 1. Therefore, *ba* was coded as −0.5, and SVO was coded as 0.5. The maximal models were fitted, and random slopes were simplified until the models converged [48]. We also provided the formula that resulted in the convergence results. The ‘other’ responses were excluded automatically and treated as N/A by R. They were not considered in the analysis of the results of the priming effects. The *MuMIn* package in R was used to calculate two types of R^2^ (marginal and conditional R^2^) to show the effect sizes of each model [51,52,53].

Table 2 indicates that order was added as a factor in the analysis of lax coding because this addition made the results convergent, although the effects associated with it were not significant. Since effects coding was applied for the analysis, the significant effect for the intercept was interpreted as a reliable syntactic priming effect. The reliable effect of Structure 1 suggested that the syntactic priming effect was stronger for the SVO than the *ba* prime when lax coding was applied. The theoretical *R*^2^_GLMM*(m)*_ of the model was 0.0933, and the theoretical *R*^2^_GLMM(*c)*_ was 0.1086, suggesting that both fixed and random effects explain relatively little of the variability in syntactic structures that children use.

When strict coding was considered, a similar pattern was obtained, except that the effect of the SVO prime was marginally more significant than its *ba* counterpart when both structures were inflected with C(omplement) and the postverbal -*le*. The theoretical *R*^2^_GLMM(*m*)_ of the model was 0.0039, and the theoretical *R*^2^_GLMM(*c)*_ was 0.0423, suggesting that both fixed and random effects explain relatively little of the variability in syntactic structures that children use.

In summary, when both structures were inflected with *Cle*, reliable syntactic priming was demonstrated in two ways of coding, but the SVO structure tended to result in stronger syntactic priming effects than its *ba* counterpart.

Table 3 indicates a similar pattern when the prime structures were inflected with the postverbal -*le* alone that we obtained when both prime structures were suffixed with *VC-le* in lax coding. Both prime structures together demonstrated a reliable syntactic priming effect, as indicated by the significant intercept effect, although the effect was stronger for the SVO priming effect than for the *ba* priming effect. The theoretical *R*^2^_GLMM(*m*)_ of the model was 0.1407, and the theoretical *R*^2^_GLMM(*c)*_ was 0.2720, suggesting that both fixed and random effects explain relatively little of the variability in syntactic structures that children use.

However, the results from the strict coding painted quite a different picture regarding the priming effect. The significant but negative estimate and z value in the intercept suggested that nonprime trials were produced more significantly than the primed trial was, but the SVO prime structure still led to a stronger prime effect than its *ba* counterpart was. With reference to the patterns shown in the figure above, we suggest that *ba* priming when inflected with -*le* leads to too few primed responses, i.e., nonprimed responses, resulting in the pattern that we observed. The theoretical *R*^2^_GLMM(*m*)_ of the model was 0.4697, and the theoretical *R*^2^_GLMM(*c)*_ was 0.5367, suggesting that both fixed and random effects explain relatively a medium level of the variability in syntactic structures that children use.

In summary, when both of the structures were inflected with -*le*, syntactic priming with both structures was obtained only when lax coding was applied. When strict coding was applied, only the SVO structure, not the *ba* structure, led to reliable syntactic priming. Regardless of the coding methods used and the occurrence of reliable syntactic priming, the SVO structure resulted in more syntactic priming effects than its *ba* counterpart.

Table 4 indicates that although a general priming effect did not exist for both primes because the intercept was not reliable, the effect of the SVO prime was still significantly stronger than that of the *ba* prime in the lax coding. When strict coding was applied to the experiment in which both primes were inflected with the complement only, namely, C, nonprimed trials were produced significantly more often than primed trials. Nevertheless, primed trials were exhibited significantly more often with the SVO prime than with the *ba* prime. The theoretical *R*^2^_GLMM(*m*)_ of the model was 0.1279 for lax coding and 0.0236 for strict coding, and the theoretical *R*^2^_GLMM(*c)*_ was 0.3183 for lax coding and 0.3574 for strict coding, suggesting that both fixed and random effects in both coding methods explain relatively little of the variability in syntactic structures that children use, but both effects explain more variability in syntactic structures when strict coding is applied.

In summary, when both structures were inflected with *C*, no reliable syntactic priming was obtained by coding schemas. Nevertheless, the SVO structure still resulted in more primed SVO utterances than its *ba* counterpart.

To analyze the combined data, we coded the variable of Structure at −0.5 for *ba* and 0.5 for SVO and applied orthogonal contrast to code the variable of form *VC-le*, -*le*, and *VC* using the pair of contrasts of (0, −0.5, 0.5) and (2/3, −1/3, −1/3) so that the primed trials were compared between the forms of -*le* and *VC* and among the three forms.

Table 5 indicates that the combined results of lax coding indicated a pattern similar to that obtained from the three separate subexperiments with lax coding. There was a general effect of syntactic priming for both prime structures, and the effect was stronger for the SVO prime than for the *ba* prime. Priming effects were stronger when both of the structures were inflected with -*le* than with *VC*, and the summation of the priming effects of the -*le* and *VC* forms was greater than that of the form of *VC-le* alone. The theoretical *R*^2^_GLMM(*m*)_ of the model was 0.1403, and the theoretical *R*^2^_GLMM(*c)*_ was 0.2953, suggesting that both fixed and random effects explain relatively little of the variability in syntactic structures that children use.

The results of the strict coding revealed a somewhat different pattern than did the lax coding. First, more nonprimed trials than primed trials were produced, as suggested by the negatively significant intercept effect. Although the SVO prime seemed to lead to more primed trials than the *ba* prime, inflecting both prime structures with -*le* priming did not necessarily result in more prime trials than when they were inflected with *VC priming.* Their effects must be evaluated in terms of the significant interaction effects. The theoretical *R*^2^_GLMM(*m*)_ of the model was 0.3110, and the theoretical *R*^2^_GLMM(*c)*_ was 0.4106, suggesting that both fixed and random effects explain relatively a medium level of the variability in syntactic structures that children use.

Table 6 indicates that although *ba* priming typically led to smaller priming effects than SVO priming did, this difference was somewhat mitigated when both prime structures were inflected with *VC-le*. The difference between the two prime structures was marginally significant (*p* = 0.0820). On the other hand, the two prime structures resulted in unbalanced priming effects on the basis of the inflectional forms that were attached to them. The SVO form with the *postverbal -le* created the most effective prime of the three, and the *VC-le* form was more effective than the *VC* alone. However, *ba* with the *VC-le* form created the most effective prime of the three, and *ba* with the *VC* form was more effective than ba with the postverbal -*le*. In other words, to exhibit reliable syntactic priming effects, the two prime structures seem to require different postverbal inflectional forms.

## 4. Discussion

When different inflections are attached to the verbs in the SVO and *ba* constructions, variations in these inflections result in different levels of syntactic priming within a VP in Mandarin-speaking five-year-olds. With lax coding, which considers only whether the SVO or *ba* constructions are produced regardless of the postverbal inflections, any of the inflections attached to the verbs in the SVO construction serve as an effective prime structure to induce syntactic priming. On the other hand, not all three inflections served as effective primes in the *ba* construction as their SVO counterparts. When the complement plus -*le*, namely, *VC-le* and postverbal -*le* but not the complement alone, was attached to the verbs in the *ba* construction, it served as an effective prime structure for subsequent syntactic priming.

However, when strict coding that requires the produced form is needed to mimic the prime structures, a clear three-way contrast for the SVO-*ba* alternation emerges. When the verbs of both structures are inflected with the complement and the post verbal -*le*, they serve as effective prime structures to induce syntactic priming. While the verbs in the SVO construction inflected with -*le* serve as an effective prime structure, they are not effective prime structures when they are inflected with the complements alone. In contrast, when the verbs in the *ba* construction are inflected with either -*le* or the complement alone, neither of these two types of inflections serve as effective prime structures that induce reliable syntactic priming.

The following discussion employs the three-way contrast of (un)reliable syntactic priming with respect to the two structures in the SVO-*ba* alternation found in the current study. This discussion elucidates issues of the linguistic representation of complements and -*le* that follows verbs in Mandarin Chinese, levels of linguistic representation that syntactic priming may target during language production, and issues regarding syntactic priming in psycholinguistic and linguistic theories may be resolved by future neurolinguistic studies cross-linguistically.

### 4.1. What Are Linguistic Representations of the Postverbal Complement and -le?

The statistical results reported earlier indicated a three-way contrast of the three inflectional forms for both of the prime structures investigated in the current study, although not all of them served as an effective prime to induce syntactic priming. They are better treated as different syntactic representations that project their own phrasal structures because morphological elements such as tense and aspect are less likely to lead to different magnitudes of syntactic priming [22]. As a result, analyses of the inner representation as syntactic rather than morphological for *VC* gain ground [8,9,10]. Sybesma’s analyses of postverbal -*le* with its own syntactic projection [8,9] are supported. However, analyses of the complement in the VC as a morphological element within a compound [3,4,5] and of the postverbal -*le* as a suffix attached to the verb [1,2,43] do not gain support from the results.

The contrasting results of the inflectional forms also suggest some interesting explanations for how Mandarin-speaking five-year-olds may use certain specific grammatical representations during language production through syntactic priming. We specifically allowed the postverbal -*le* to be ambiguous between the realization -*le* and the completion/end point -*le*. Children’s different productions of the postverbal -*le* with respect to the SVO and *ba* constructions suggest that when -*le* is attached to the verb alone in the two structures, their grammatical representations in production are tuned to the realization -*le* [5] but not the completion -*le*. Since the SVO structure imposes a semantics of realization, when this semantics is met, priming effects of the verb plus the postverbal -*le* occur. This imposition is also compatible with the postcomplement -*le* in the SVO structure as -*le* telicizes the resultative complement to a realized state. In contrast, VPs with complements only denote resultative semantics but lack assured realization of the event, blocking the occurrence of syntactic priming [9].

Children’s inability to demonstrate syntactic priming in the type of *ba* construction inflected by -*le* alone suggests that the postverbal -*le* might retain its realized function and does not further convert it to completion in their production, as opposed to Sybesma’s argument [9]. Nevertheless, the reliable syntactic priming that was obtained when *ba* was inflected with -*le* in lax coding suggests that resultativeness is key for the role of the *ba* construction [4,40,41,42] because this type of *ba* construction, namely, *V + le*, broadly primes the *ba* construction with a resultative complement either with or without -*le* attached to it. This type of *ba* construction primes the *ba* construction such that resultativeness should be present, and its following must telecize the event to a state [8]. Therefore, while the final stage of production requires children to recheck the status of -*le* so that they can squeeze the resultativeness back and force it to a coerced form, incorporating resultativeness, telicity, and completion seems least likely. With this speculation in mind, in addition to the findings that both structures inflected with *VC-le* led to reliable syntactic priming, the inabilities of both prime structures inflected with complements suggest that different grammatical representations may be required for the two prime structures [7]. For the SVO structure to be an effective prime, realization of the event seems to matter most; therefore, as long as -*le* is attached, this level of representation guarantees reliable syntactic priming. In contrast, although resultativeness is key for *ba* construction, it does not seem sufficient to induce syntactic priming because *VC-le*, but not *VC*, led to reliable priming. However, it serves as an intermediate representation, as we can glean from the contrast of the reliable syntactic priming effects in the lax coding and unreliable ones in the strict coding when the *ba* prime structure is inflected with -*le* alone as well as more syntactic priming effects when the prime structure is inflected with *VC* than when it is inflected with -*le* alone.

### 4.2. How Many Levels of Syntactic Representations Does Syntactic Priming Target?

Following Bock and colleagues’ theoretical claims [46], Pickering and colleagues [12,22,26] claimed that there is one single syntactic representation to which syntactic priming is sensitive. However, Hartsuiker and Westenberg’s [20] and Konopka and Bock’s [21] findings that syntactic priming is sensitive to word order variation with the least semantic interference within a verb phrase suggest that there is at least one syntactic representation within VPs in addition to the clause as a whole. Furthermore, the three-way contrast of the types of inflections investigated in the SVO construction suggests that two levels of phrasal representations, as Sybesma has argued [9]. There is one grammatical representation for the (C)omplement and one for the -*le*, contrary to the claim that the subverbal elements did not project their own grammatical representations [22] that syntactic priming is sensitive to.

Although Pickering et al. [26] drew on the negative evidence of the inability of a shift-dative to induce reliable syntactic priming, this negatively based evidence can only state that there are other, stronger factors (e.g., frequency) [12] that prevent the structure from being an effective prime for syntactic priming. The three-way contrast of the same types of inflections attached to the verbs in the *ba* construction seems to paint a somewhat similar story, particularly for the *VC* inflection. Their inability to function as effective primes may be due to some participants’ uneasy use of this type of *ba* construction, as in the English shift-dative [12,26], which is subject to constraints that may be outside grammar. Huang and Yang [38] explicitly stated that this type of grammatical construction is unacceptable. Nevertheless, grammatically speaking, at least in terms of the norming study [39] or reported cases in the literature [9], the grammatical conditions and representations of this type of prime should lead to reliable syntactic priming, but individual variations of comfortable usage of this structure may block its production.

Chang et al.’s [27] syntactic priming as a learning account inspired many developmental psycholinguists to investigate how various structures with different structure frequencies may impact syntactic representations, leading to syntactic priming among preschoolers. Although many of the findings for young children did not fully conform to predictions [33,34,37], Kumarage et al. [31] reported that English-speaking 3-year-olds demonstrated greater syntactic priming effects than their older counterparts did; younger children may need more error adjustments to their developing syntactic representations with the passive structure so that greater syntactic priming is induced. Mandarin-speaking five-year-olds’ sensitivities to syntactic priming in terms of the three types of inflections attached to the *ba* construction seem to follow Chang et al.’s [27] predictions. Du [54] asked native adults to describe pictures that involved transitive events and reported that among all of the utterances produced via *ba* constructions, the *VC* form had the highest production rate, followed by the -*le* form and then the *VC-le*. The results suggest that when *ba* constructions are inflected with VC, they are commonly produced proportionally to Huang and Yang’s [38] claim. If the greatest ‘other’ utterances in the *VC* form are included for the frequency counts, the *ba* construction in this inflectional form may be subject to more constraints in production [38]. Chang et al.’s [27] implicit account explains why *VC-le* would be the inflection most likely to be primed, followed by *VC* and then -*le*. On the other hand, the finding that the less commonly used *ba* construction tends to induce stronger priming effects was also reported, but only when the *ba* prime structure was inflected with both the complement and the postverbal -*le* [36,37]. This account does not seem to fit well with the data with respect to the SVO construction. It is speculated that the error adjustment to this construction is less likely and that grammatical representations associated with each of the inflectional forms play a greater role than frequency does.

### 4.3. Neurolinguistics as an Alternative Way to Investigate Grammatical Representations

Saffran and Martin [55] and Hartsuiker and Kolk [56] used a paradigm similar to what was employed in the current study but asked the aphasia participants of whom the former study investigated five participants with various aphasia subtypes and the latter specifically with twelve Broca’s aphasia participants to repeat the prime and then describe the targets with one sentence [13]. Both studies revealed that aphasic participants were able to exhibit syntactic priming with the passive voice but not the active voice. The set of results was similar to what was discussed in Kumarage et al.’s study and was consistent with Chang et al.’s syntactic priming as a learning account.

Ledoux, Traxler, and Swabb [57] used the main clause (MC) prime *the speaker proposed the solution to the group at the space program* and the reduced-relative (RR) clause primes *the speaker proposed by the group would work perfectly for the program* with verbs that are shared with the following RR target *the manager proposed by the **directors** was a bitter, old man* to investigate how comprehenders may understand RR primes via the event-related potential (ERP) technique. Their results indicated that RR primes elicited greater positivity than MC primes did, but the positivity at the critical noun shown in bold above in the RR targets was reduced significantly after the RR primes compared with the MC counterparts when the same verb was shared between the prime and the target. Segaert, Kempen, Peterson, and Hagoort [58] reported that the blood-oxygen-dependent (BOLD) response detected by fMRI was adapted for syntactic priming with the passive voice but not for the active voice in the left inferior frontal gyrus (IFG) and posterior middle temporal gyrus (MTG). Such adaptation occurred in both comprehension and production similarly, even when the verbs were not shared between the prime and the targets. They argued that syntactic priming could occur in comprehension without necessarily appealing to the overlap of lexical heads in prime and target. It is the passive voice that is much less frequent, so a priming benefit suppression effect could appear more readily to be detected by BOLD.

However, the results obtained in the current study were quite different from those reported in the neurolinguistic and psycholinguistic studies above. Hagoort [59] suggested that issues related to syntactic priming can be investigated in three ways, i.e., linguistically, psycholinguistically, and neurolinguistically, to better understand the underlying workings of human minds with respect to linguistic representations. The apparent discrepancy in the results of the current study from English-based results calls for future cross-linguistic exploration to resolve the issues raised here. First, the SVO construction in Mandarin Chinese is the predominant structure and has a much higher frequency than the *ba* construction does. If we follow the results reported particularly on neurolinguistic evidence, then the *ba* structure should stimulate a greater BOLD response than its SVO counterparts, but the results with syntactic priming in the current study indicate the other way around. This may imply a dilemma in the presented explanation because low-frequency constructions elicit a stronger BOLD response, which is detected by the priming benefit suppression effect. Notably, we do not claim that syntactic priming in Mandarin Chinese involves dramatically different regions other than the left IFG and MTG in the brain. Sakai [60] reported that bilinguals engaged in similar cerebral regions, such as the left IFG, when conducting tasks involving tense formation in verbs versus verb matching in both English and Japanese. His results suggested that there was a universal grammar center involved in grammatical operations. Nevertheless, it is unclear how a much more common construction that leads to a greater syntactic priming effect than its much less common counterparts can be represented in the brain in terms of BOLD or EEG positivity, as they may also highlight cross-linguistic differences. Second, the rationale behind the less common passive voice and greater BOLD activation may rely on the assumption that the less common the structure is, the more difficult it is to process. However, Ferreira [61] argued that it is the thematic arrangement of the order of agent–patient but not the frequency that affects the assumed difficulty. In other words, when the structures conform to the thematic arrangement of agent‒patient order, such as the English active voice, as well as the much less common subject cleft construction, they can be processed similarly, which in turn are processed significantly better than the passive voice and object cleft constructions, which have a reversal arrangement of the thematic order of patient‒agent. As the SVO-*ba* alternations both have the thematic arrangement of agent–patient, they are processed similarly in accuracy [62,63] and response times (RTs) [63]. Nevertheless, Hsu [62] reported that there is an age effect on Mandarin preschoolers’ development in terms of the order of thematic roles, whereas such an age difference with syntactic priming does not exist [37]. This unstable age effect on children’s syntactic development has also been reported in English-based studies [29,30,31,32,33,34]. Neurolinguistic investigations cross-linguistically help illuminate the effects of age, structural type, or methodology. Third, it is tightly locked to the research theme in the current study. Kielar, Milman, Bonakdarpour, and Thompson [64] used temporal words such *Yesterday* and *Nowadays* to elicit past tense and agreement-third person singular present tense that are inflected in the verb in healthy adult participants. They reported that although similar regions, such as the left IFG and motor and premotor cortices, were recruited for the two forms, distinct frontal and parietal regions were also recruited. Furthermore, the regions recruited to elicit the verb stem were different only from the two forms inflected with tense. Their results suggested that independent grammatical representations can be constructed for sublexical components. As a result, it is likely that the postverbal complement and -*le* can also have shared but still distinguishable representations in the brain. Neurolinguistic investigations comparing different structural types as well as sublexical operations within a construction cross-linguistically, which are critical to theoretical inquiries concerning not only syntactic priming but also entire cognitive science as a whole if the inner workings of human minds about language need to be fully understood.

## 5. Conclusions

The current study employed syntactic priming of the SVO-*ba* alternation to investigate the linguistic debate about grammatical representations of the complement that follow the verb and of the postverbal -*le* that follow the complement, namely, *VC-le*, or follow the verb directly, namely, the postverbal -*le* within the VP. The results suggest that VC should be represented in terms of a VP instead of a verb compound, and the postverbal -*le* should function as a predicate and project its own phrasal structures. Mandarin-speaking 5-year-olds demonstrated different levels of magnitude in syntactic priming with respect to these structures. The report of grammatical forms that are able and unable to induce reliable syntactic priming in the current study challenges Pickering and Branigan’s [22] single lexico-syntactic representation account and Chang et al.’s [27] implicit learning account, both of which claim that syntactic representations that are sensitive to syntactic priming are modality neutral. Even if grammatical representations are well-formed in comprehension through judgments of grammaticality, not all grammatically well-formed representations are necessarily those to which syntactic priming is sensitive and therefore ready for subsequent production. The results also suggest that there may be a discrepancy of the same syntactic representations to be drawn upon in comprehension and production. Nevertheless, the study also highlights several important aspects that the current mainstream accounts for syntactic priming cannot fully explain and supports the position that neurolinguistic techniques can serve as promising tools to pursue cross-linguistic inquiries with respect to issues intertwined among linguistics, psycholinguistics, and neurolinguistics in cognitive science.

## Figures and Tables

**Table 1:** Children’s response counts with percentages in parentheses in each priming condition across two types of coding schemas.

Forms	Prime	Loose			Strict		
		SVO	BA	Other	SVO	BA	Other
VC-le	SVO	555 (64.2%)	244 (10.98%)	214 (24.74%)	394 (45.60%)	256 (29.63%)	214 (24.77%)
	BA	95 (28.24%)	446 (51.62%)	174 (20.14%)	311 (36.00%)	379 (43.87%)	174 (20.14%)
-le	SVO	582 (67.36%)	106 (12.27%)	176 (20.37%)	480 (55.56%)	208 (20.07%)	176 (20.37%)
	BA	284 (32.87%)	436 (50.46%)	144 (16.67%)	668 (75.62%)	52 (6.47%)	144 (17.91%)
VC	SVO	426 (39.31%)	127 (14.70%)	311 (36.00%)	177 (20.49%)	376 (43.52%)	311 (36.00%)
	BA	285 (32.99%)	286 (33.10%)	293 (33.91%)	453 (52.43%)	118 (13.66%)	293 (33.91%)

**Table 2:** Mixed logit model results for the *VC-le* results in the two coding schemas.

Contrast	Estimate	SE	z Value	Pr (>|z|)
Lax coding				
Formula: Lax coding~1 + Structure*order + (1 + Structure|subject) + (1|item), data = VC-le_results, family = binomial				
Intercept	**1.1807**	**0.1083**	**10.900**	**<0.01**
Structure 1 (SVO vs. BA)	**1.0923**	**0.2166**	**5.043**	**<0.01**
Order 2 (SVO vs. BA)	0.0176	0.1380	0.128	n.s
Structure1:Order2	0.1561	0.3384	0.461	n.s.
Strict coding				
Formula: Strict coding~1 + Structure + (1 + Structure|subject) + (1|item), data = VC-le_results, family = binomial				
Intercept	**0.3220**	**0.0750**	**4.291**	**<0.05**
Structure 1	***0.2313***	***0.1227***	***1.885***	***0.0594***

**Table 3:** Mixed logit model results for the -*le* results in the two coding schemas.

Contrast	Estimate	SE	z Value	Pr (>|z|)
Lax coding				
Formula: Lax coding~1 + Structure + (1 + Structure|Subject) + (1|item), data = le_results, family = binomial				
Intercept	**1.2390**	**0.0980**	**12.639**	**<0.01**
Structure 1 (SVO vs. BA)	**1.5944**	**0.2281**	**6.989**	**<0.01**
Strict coding				
Formula: Strict coding~1 + Structure + (1 + Structure|Subject) + (1|item), data = le_results, family = binomial				
Intercept	**−0.8733**	**0.1338**	**−6.529**	**<0.01**
Structure 1	**3.6522**	**0.2435**	**14.998**	**<0.01**

**Table 4:** Mixed logit model results for the *VC* results in the two coding schemas.

Contrast	Estimate	SE	z Value	Pr (>|z|)
Lax coding				
Formula: Lax coding~1 + Structure + (1 + Structure|subject) + (1|item), data = VC_results, family = binomial				
Intercept	0.0086	0.1437	0.060	n.s.
Structure 1 (SVO vs. BA)	**1.5708**	**0.2422**	**6.485**	**<0.01**
Strict coding				
Formula: Strict coding~1 + Structure + (1 + Structure|subject) + (1|item), data = VC_results, family = binomial				
Intercept	**−1.7813**	**0.2240**	**−7.953**	**<0.01**
Structure 1	**0.6947**	**0.2582**	**2.690**	**<0.01**

**Table 5:** Mixed logit model results for the combinations of the three experiments in the two coding schemas.

Contrast	Estimate	SE	z Value	Pr (>|z|)
Lax coding				
Formula: Lax coding~1 + Structure*form + (1 + Structure|subject) + (1|item), data = X3_exps_combined, family = binomial				
Intercept	**1.1381**	**0.0652**	**17.453**	**<0.01**
Structure 1 (SVO vs. BA)	**1.5381**	**0.1401**	**10.979**	**<0.01**
formleVSVC	**−0.4669**	**0.1476**	**−3.164**	**<0.01**
formVCLevsTwo	**−0.3906**	**0.1303**	**−2.998**	**<0.01**
Structure1:formleVSVC	−0.0242	0.3194	−0.076	n.s.
Structure1:formVCLevsTwo	0.0372	0.2803	0.133	n.s.
Strict coding				
Formula: Strict coding~1 + Structure*form + (1 + Structure|subject) + (1|item), data = X3_exps_combined, family = binomial				
Intercept	**−1.2969**	**0.0841**	**−15.415**	**<0.01**
Structure 1 (SVO vs. BA)	**1.4937**	**0.1051**	**14.215**	**<0.01**
formleVSVC	**−1.2198**	**0.1981**	**−6.158**	**<0.01**
formVCLevsTwo	**−2.2684**	**0.1455**	**−15.595**	**<0.01**
Structure1:formleVSVC	**−2.9648**	**0.2677**	**−11.077**	**<0.01**
Structure1:formVCLevsTwo	**1.8363**	**0.2085**	**8.807**	**<0.01**

**Table 6:** Mixed logit model results for the interaction of the models in the strict coding for the combined experiments.

Contrast	Estimate	SE	z Value	Pr (>|z|)
Pair contrasts in terms of ‘Structure’				
form = le				
BA-SVO	**−3.588**	**0.203**	**0.060**	**<0.01**
form = VC				
BA-SVO	**−0.623**	**0.181**	**−3.449**	**<0.01**
form = VCle				
BA-SVO	***−0.269***	***0.155***	***−1.739***	***0.0820***
Pair contrasts in terms of ‘form’				
Structure = BA				
le-VC	**−1.22**	**0.198**	**−6.158**	**<0.01**
le-VC-le	**−2.88**	**0.190**	**−15.135**	**<0.01**
VC-VC-le	**−1.66**	**0.161**	**−10.332**	**<0.01**
Structure = SVO				
le-VC	**1.75**	**0.183**	**9.515**	**<0.01**
le-VC-le	**0.44**	**0.175**	**2.522**	**<0.05**
VC-VC-le	**−1.30**	**0.181**	**−7.206**	**<0.01**

## Data Availability

The data presented in this study are available on request from the corresponding author due to privacy reasons.

## Appendix A

1. Tù: gēgē tù chūkǒu shuǐ.
  ‘The older brother spits out saliva.’
2. Wèn: lǎobǎn wèn wán wèntí.
  ‘The boss finishes asking the question.’
3. Gài: gōngrén gài hǎo fángzi.
  ‘The worker finishes building the house.’
4. Jiàn: huángdì jiàn hǎo wánggōng.
  ‘The emperor completes the construction of the palace.’
5. Tōu: línjū tōu dào píngguǒ.
  ‘The neighbor steals an apple.’
6. Zhuā: xiǎohái zhuā dào jīmù.
  ‘The child catches the building blocks.’
7. Jiā: chúshī jiā wán niúnǎi.
  ‘The chef adds the milk.’
8. Jiē: jiàoliàn jiē zhù lánqiú.
  ‘The coach catches the basketball.’
9. Jiè: xuéshēng jiè zǒu kèběn.
  ‘The student borrows the textbook.’
10. Jiào: lǎoshī jiāo wán yīngwén.
  ‘The teacher finishes teaching English.’
11. Xuǎn: jiějiě xuǎn cuò ěrhuán.
  ‘The older sister chooses the wrong earrings.’
12. Shōu: māmā shōu zǒu bēizi.
  ‘The mother takes away the cup.’
13. Tī: xióngmāo tī zǒu píqiú.
  ‘The panda kicks away the ball.’
14. Ná: hémǎ ná zǒu xiānhuā.
  ‘The hippopotamus takes the flowers.’
15. Tuī: lǎohǔ tuī zǒu shítou.
  ‘The tiger pushes away the stone.’
16. Jiǎn: shīzi jiǎn duàn shùzhī.
  ‘The lion cuts the branch.’
17. Diū: mèimei diū diào dàngāo.
  ‘The younger sister throws away the cake.’
18. Bào: nánhái bào zhù lánqiú.
  ‘The boy hugs the basketball.’
19. Shāo: hóuzi shāo diào zhàopiàn.
  ‘The monkey burns the photo.’
20. Sī: xiǎo gǒu sī diào bàozhǐ.
  ‘The puppy tears up the newspaper.’
21. Chuān: māomī chuān shàng yīfú.
  ‘The cat puts on clothes.’
22. Kǎo: huāpào kǎo hǎo zhāngyú.
  ‘The leopard roasts the octopus.’
23. Hē: tùzǐ hē diào guǒzhī.
  ‘The rabbit drinks the juice.’
24. Zhāi: xīngxing zhāi zǒu pútao.
  ‘The gorilla picks the grapes.’
